# No differences in visual theory of mind abilities between euthymic bipolar patients and healthy controls

**DOI:** 10.1186/s40345-016-0061-5

**Published:** 2016-10-12

**Authors:** Silvia Haag, Paula Haffner, Esther Quinlivan, Martin Brüne, Thomas Stamm

**Affiliations:** 1 Department of Psychiatry and Psychotherapy, Charité University Hospital, Charitéplatz 1, 10117 Berlin, Germany; 2 Division of Cognitive Neuropsychiatry and Psychiatric Preventive Medicine, Department of Psychiatry, Psychotherapy, and Preventive Medicine, LWL University Hospital Bochum, Ruhr University Bochum, Alexandrinenstr. 1-3, 44791 Bochum, Germany

**Keywords:** Bipolar disorder, Euthymia, Theory of mind, Social cognition, Neurocognitive functioning

## Abstract

**Background:**

Research on theory of mind (ToM) abilities in patients with bipolar disorder has yielded conflicting results. Meta-analyses point to a stable moderate impairment in remitted patients, but factors such as subsyndromal symptoms, illness severity, and deficits in basic neurocognitive functions might act as confounders. Also, differences in deficits depending on task area (cognitive or affective) or task modality (visual or verbal) have been observed. This study aimed to test the hypothesis that euthymic bipolar patients would perform more poorly than healthy subjects on visual cognitive and visual affective ToM tasks. Furthermore, we aimed to explore the relationship between ToM performance and basic neurocognitive functions, subsyndromal symptom severity, and illness burden. Twenty-nine clinically stable outpatients with bipolar disorder and 29 healthy comparison subjects completed a measure of visual cognitive ToM (Mental State Attribution Task, MSAT), a measure of visual affective ToM (Reading the Mind in the Eyes Test, RMET), and a battery of tests assessing neurocognitive functioning (attention, verbal memory, executive functions, and intelligence).

**Results:**

Patients did not differ significantly from healthy controls for the ToM tasks or any of the neurocognitive measures, suggesting a high level of neurocognitive functioning in the bipolar group. On average, patients were slower than controls to complete the ToM tasks. Within the bipolar group, ToM performance was moderately correlated with attention, verbal memory and reasoning abilities. Performance on the RMET was positively correlated with clinician-rated depressive symptoms with a small effect. Number of years of illness was weakly and negatively correlated with performance on the MSAT. Overall, no moderate or strong correlations were found between ToM performance, subsyndromal depressive or manic symptoms, illness duration, and number of depressive or (hypo)manic episodes. Moderate correlations between ToM performance and age were found for patients but nor for controls.

**Conclusions:**

Our findings suggest preserved visual cognitive and affective ToM abilities in euthymic bipolar patients characterized by a high level of neurocognitive functioning.

## Background

Theory of mind (ToM) is an aspect of social cognition that describes the ability to understand the feelings, thoughts and intentions of oneself and of others (Brüne 2008). The term was originally introduced in 1978 by Premack and Woodruff (1978) in an article that discussed the ability of chimpanzees to infer mental states that are not directly observable (for example, intentions, knowledge or beliefs) and that can serve to predict the behavior of others. The article sparked a wave of research on ToM that has been increasing ever since. As Schaafsma et al. (2015) point out, the original definition of ToM referred to a variety of processes which led to heterogeneity in the scientific methods approaching ToM. Thus, as research on the topic is growing, attempts to clarify the construct are advancing.

In psychological terms, ToM has recently been described as dual-process schemes that distinguish either between mental state reasoning (social-cognitive processes) and mental state decoding (social-perceptual processes) (Samamé et al. 2012) or between cognitive and affective ToM (Shamay-Tsoory et al. 2007). The identification of particular ToM processes is directly related to the interpretation of different tasks designed to measure ToM abilities. Tasks that request subjects to understand the beliefs or false beliefs of other persons to detect hidden intentions, indirect meaning, or inappropriate social communication are assumed to account for cognitive ToM or mental state reasoning. On the other hand, tasks that demand of subjects to infer the feelings or affective mental states of others are regarded as measures to assess affective ToM and also mental state decoding abilities if visual information such as facial expressions needs to be decoded. These dual-process schemes are supported by evidence from imaging studies that have reported task-specific differences in brain activation patterns (Kalbe et al. 2010; Schurz et al. 2014; Shamay-Tsoory and Aharon-Peretz 2007).

The ability to understand the mental or affective states of others has been found to be impaired in some developmental and mental disorders. ToM deficits have consistently been observed in patients with autism spectrum disorders (Frith 2012) and schizophrenia (Savla et al. 2013). Bipolar disorder (BD) is often discussed in relation to schizophrenia and much research is done comparing the two conditions. In BD, ToM deficits have been less consistently shown than in schizophrenia (Mitchell and Young 2016). Studies on ToM in BD vary widely in how ToM has been operationalized, hence rendering the collective findings difficult to interpret. This may be related to possible moderating variables such as subsyndromal symptom severity, indicators of illness burden, and basic neurocognitive functions not always being taken into account. Indeed, the association between these factors and ToM abilities has, to date, not been explicitly clarified.

Recently, two task-specific meta-analyses were conducted to synthesize the heterogeneous results yielded so far in this field. A meta-analysis by Samamé et al. (2015) reported small-to-medium effect sizes for tasks measuring cognitive ToM and small effect sizes for tasks measuring affective ToM in favor of healthy controls (HCs) versus euthymic bipolar patients (eBPs). Based on a slightly extended data pool, Bora et al. (2016) specifically investigated the relationship between ToM deficits and mood state. Pooling all studies irrespective of task modality or content, they found stable moderate effects in strictly euthymic BD samples, as well as in subsyndromal samples, favoring HCs. In acute BD samples, a strong effect favoring HCs was found, which was significantly more severe than the effect found for remitted or subsyndromal samples. ToM deficits seem to be more reliably present in acute BPs than in strictly euthymic samples (Kerr et al. 2003; Ioannidi et al. 2015). The first prospective study to assess the effect of remission on ToM performance in BPs has found patients to recover nearly all of their neurocognitive and social-cognitive abilities during remission, with the exception of performance on the Faux Pas Task, verbal memory, and visuospatial working memory (Ioannidi et al. 2015). In the meta-regression analyses performed by Bora et al. (2016), clinician-rated manic symptoms were significantly related to the severity of ToM impairment, but not clinician-rated depressive symptoms. No significant effects of gender, age, education, illness duration, or age at illness onset on ToM performance in remitted or subsyndromal BPs were found. In contrast, global cognitive impairment was significantly associated with ToM deficits in the whole sample and in remitted and subsyndromal samples only. In previous studies, ToM performance in bipolar-only and mixed samples (i.e., with both eBPs and HCs) has been found to correlate with general intellectual functioning, attention, memory and executive functions (Ioannidi et al. 2015; Bora et al. 2005; Purcell et al. 2013; Wolf et al. 2010; Martino et al. 2011; Donohoe et al. 2012). Performing a path analysis, Van Rheenen et al. (2014) found that neurocognition (a composite score) directly predicted variance in ToM performance, concluding that neurocognition and social cognition are related in BD. As ToM performance seems to correlate with a variety of neurocognitive functions, it should also be considered that BD may be characterized by consistent deficits across almost all basic neurocognitive domains (for a comprehensive review of recent data, see Fountoulakis (2015)). Deficits have most consistently been shown for executive functions (particularly response inhibition) and verbal memory, both of which have been discussed as possible cognitive endophenotypes for BD (Fountoulakis 2015).

Few studies, so far, have examined secondary measures of ToM, such as reaction times or task completion duration, and findings have conflicted. Two studies have reported eBPs to perform more slowly than HCs on an affective ToM task and a cognitive ToM task, respectively, while accuracy was the same for both groups in both studies (Kim et al. 2009; Olley et al. 2005). In contrast, another study found eBPs to show faster reaction times than HCs in a computerized affective ToM task with accuracy again being consistent (Purcell et al. 2013). In that study, quicker responding was related to lower life functioning at a 1-year follow-up.

In the present study, we aimed to specifically investigate visual cognitive and visual affective ToM abilities in eBPs and to explore their relationship with basic neurocognitive functions, subsyndromal symptom severity, and indicators of illness burden. As primary outcome measures, we chose the Mental State Attribution Task (MSAT; Brüne 2003) to measure visual cognitive ToM, and the Reading the Mind in the Eyes Test (RMET; Baron-Cohen et al. 2001) to measure visual affective ToM. Next, we were interested in the difference of time taken to complete the ToM tasks in patients versus controls.

We hypothesized that euthymic bipolar patients would perform worse than healthy controls on both the MSAT and the RMET. Further, we hypothesized that patients would differ in the time they would take to complete the tasks compared to the controls, but made no predictions about the direction of the effect. Correlations between variables were tested exploratively.

## Methods

### Design and participants

The study employed a cross-sectional correlational design to describe the abilities of euthymic bipolar patients relative to healthy controls in visual cognitive and affective ToM and to examine the relationship between ToM performance and neurocognitive functioning, subsyndromal symptom severity, and illness burden. Patients were recruited at the outpatient clinic of the Department of Psychiatry and Psychotherapy at Charité University Hospital Berlin, Campus Mitte. Patients had to be euthymic for a minimum of 6 weeks prior to testing as determined by a score ≤9 on the 21-items-version of the Hamilton Depression Rating Scale (HAMD; Hamilton 1960) and a score ≤12 on the Young Mania Rating Scale (YMRS; Young et al. 1978). Further, patients’ mood-stabilizer intake had to be stable for at least the six preceding weeks. Exclusion criteria included a history of substance abuse or electroconvulsive therapy in the last 6 months, and a previous diagnosis of schizoaffective disorder, schizophrenia, antisocial personality disorder, dementia, mild cognitive impairment or mild intellectual disability according to ICD-10 (World Health Organisation 2016). Patients were not preselected on the basis of any neurocognitive measure before entering the study. Parallel to the study, patients were invited to participate in an 8-week metacognitive training program after completing the study measures. Patients did not receive financial compensation for this. Out of 72 subjects screened, 34 patients were included in the present study. A total of five patients were excluded from analysis post hoc: Four patients revealed to meet exclusion criteria (e.g., current alcohol abuse, mild intellectual disability and acute depression), while one patient refused to participate in the cognitive ToM task and was excluded due to missing data. The final clinical sample included 29 individuals diagnosed with bipolar disorder type I or II according to ICD-10 criteria.

The control sample was recruited by online advertisement or via contacts of the researchers. Exclusion criteria included a history of any psychiatric disorder as assessed via the Mini International Neuropsychiatric Interview (MINI; Sheehan et al. 1998), self-reported severe physical health problems, and first-degree relatives with a psychiatric condition. Thirty-four subjects were included in the study. Five participants were excluded post hoc from analysis, because they had a history of an affective disorder or substance abuse. The final sample comprised 29 healthy participants. The study was approved by the local ethics committee. All participants provided written informed consent.

## Materials

### Clinical measures

In patients, current depressive symptoms were measured via the HAMD and the Beck Depression Inventory (BDI; Beck et al. 1961) Manic symptoms were assessed with the YMRS and the Altman Self-Rating Mania Scale (ASRM; Altman et al. 1997). Also, duration of illness and number of previous depressive and (hypo)manic episodes were recorded as demographics. Controls were administered the MINI, the BDI, and the Brief Symptom Inventory (BSI; Franke 2000).

### Theory of mind tasks

Visual cognitive ToM was assessed with the Mental State Attribution Task (MSAT) developed by Brüne (2003). This task consists of six ToM cartoon stories, each comprising four picture cards. For each story, the four cards were placed face down in front of the participants in random order. The participants were then requested to turn them over and organize the cards into a logical sequence. The time taken for sequencing was noted. If the cards were sequenced incorrectly, the experimenter placed them in the right order. Next, the participants were asked questions about the mental states of the characters in the cartoon. To answer the questions correctly, the participants had to understand characters’ first-order, second-order, or third-order true or false beliefs, reciprocity, deception and cheating intentions. Two questions were “reality” questions that did not require inferences about mental states but rather merely the detection of facts from the story that were visually comprehendible. Two of the picture stories as well as the method of rating were adapted from Langdon et al. (1997). The correct sequencing of the first and last cards was given two points, while the correct sequencing of the second and third card was awarded one point. Each correctly answered question was awarded one point. A total score comprising the sequencing-accuracy score and the correctly answered questions was computed (with a maximum of 59 points). Two practice samples depicting “reality” (i.e., non-ToM) stories taken from Langdon et al. (1997) were administered before the main task. The variables of interest were the total score and the total time taken for the sequencing of the six cartoons (measured in seconds).

Visual affective ToM was measured with the Reading the Mind in the Eyes Test (RMET; German version by Bölte (2005)). The RMET consists of a series of 36 sheets with a pair of eyes printed in the middle of each sheet. Each pair of eyes is presented with four words that describe emotions or affectively valenced states of mind (e.g., “anxious” or “confused”). From an answer sheet, the participants were instructed to choose the word that best described what the person in the picture was feeling, thinking or trying to express. No time restrictions were imposed, but participants were informed that they were being timed. A maximum of 36 points could be achieved. The variables of interest were the total score and the time taken to complete the task measured in minutes.

### Neuropsychological measures

The neuropsychological assessment comprised 17 tests measuring selected facets of attention, memory, executive functions, and general cognitive ability. Three subtests of the computer-based Test of Attentional Performance (TAP; Zimmermann and Fimm 1994) were administered to measure tonic and phasic alertness (subtest “Alertness”, reaction time), visual and auditory divided attention (subtest “Divided Attention”, reaction time), general divided attention (subtest “Divided Attention”, number of lapses), and selective attention (subtest “Go/No-go”, reaction time). Auditory short-term memory and auditory working memory were measured using the Digit Span Forward and Digit Span Backward subtests of the revised Wechsler Memory Scale (WMS-R; Härting et al. 2000). The Verbal Learning and Memory Test (VLMT; Helmstaedter et al. 2001) was used to assess verbal learning, verbal consolidation and verbal recognition. Cognitive inhibition was measured with the Go/No-go subtest of the TAP (number of errors) and with the Stroop Colour–Word Task (Bäumler 1985). Phonemic and semantic verbal fluency were measured with the subscales “S-words” and “animal names” of the Regensburger Wortflüssigkeits-Test (RWT; Aschenbrenner et al. 2000). The third subtest of the German intelligence test battery Leistungsprüfsystem (LPS3; Horn 1983), a figure sequence task, was used to assess reasoning abilities. Verbal intelligence was measured with version B of the Mehrfachwahl-Wortschatz-Intelligenztest (MWTB; Lehrl 2005) which is similar to the Spot-the-Word Test (Baddeley et al. 1993).

All tests and interviews were conducted by trained postgraduate psychology or medical students.

### Statistical analyses

Data were analyzed with RStudio Desktop version 0.99.484 (R Core Team 2013). To determine group differences between eBPs and HCs, several statistical procedures were employed depending on the scale of variables and their distributions. Group comparisons between categorical variables were performed using the Chi-squared test. The distributional properties of each continuous variable were graphically explored using histograms and box plots. For continuous variables, data outliers were identified and the assumptions of normality were tested using the Shapiro–Wilk test for each group level separately. Group differences on variables that were normally distributed and did not contain outliers were analyzed using independent samples *t* tests. Welch’s adjustment was set as the default. Differences on variables that were not normally distributed or contained outliers in one or both of the groups were analyzed with the Yuen–Welch *t* test (Yuen 1974), a robust test to compare independent means that employs trimmed population means and Winsorized variances in combination with Welch’s two-sample test. The level of trimming in our study was set to 20 %.

Two measures of effect size were computed for group comparisons on neurocognitive and ToM variables: Cohen’s *d,* and the *explanatory measure of effect size ξ* (xi) proposed by Wilcox and Tian (2011). Xi is a robust measure of effect size that allows for heteroscedasticity. Cohen’s *d* is based on the mean and variance, which means that it is not robust when the assumptions of normality and homoscedasticity are not met. Conventionally, *d* = 0.2, 0.5 and 0.8, as well as *ξ* = 0.15, 0.35 and 0.50 are considered small, moderate and large effect sizes (Cohen 1988; Wilcox 2012). To facilitate the comparability of our results to other studies, we decided to report results of Welch’s *t* test and Cohen’s *d* also for variables that did not fulfill the aforementioned assumptions. Nonetheless, inferences were based on robust tests for variables that presented non-normal distributions or outliers.

Kendall’s tau correlation coefficient, *τ*, was calculated as a robust measure to determine associations between variables. Conventionally, correlations around 0.10, 0.30 and 0.50 are considered weak, moderate and large effect sizes, respectively (Cohen 1988). Furthermore, a minimum correlation of 0.20 is recommended as a marker of practical significance (Ferguson 2009); henceforth, only correlations above that level are reported and discussed.

The alpha-level was set to 0.05 for all statistical tests. When multiple tests were conducted, Bonferroni-adjusted alpha levels were used for hypothesis testing.

## Results

### Demographic and clinical features

Patients and controls were matched for gender and years of education. On average, patients were older than controls and this difference was statistically significant. Demographic and clinical features of the samples are displayed in Table 1.

**Table 1:** Participant characteristics

Measure	Bipolar patients		Healthy controls		Statistics	*df*	*p*
	*n*		*n*				
Age^a^, *M* (SD)	29	47.8 (13.6)	29	40.8 (12.7)	*t* = 2.009	55.73	.049
Years of education, *M* (SD)	29	15.0 (2.4)	29	14.8 (2.8)	*t* = 1.215 *t*_y_ = 1.222	55.12 34.27	.230 .230
Gender, male/female ratio	29	16/13	29	10/19	*χ* ^*2*^ = 1.743		.187
BDI score, *M* (SD)	29	10.9 (8.9)	29	3.6 (3.9)	*t* = 4.001 *t*_y_ = 3.077	36.96 21.57	<.001 .006
ASRM score, *M* (SD)	29	2.3 (2.6)					
HAMD score, *M* (SD)	29	5.7 (2.8)					
YMRS score, *M* (SD)	29	1.5 (2.2)					
Bipolar disorder type I, *n* (%)	29	15 (52)					
History of psychotic symptoms, *n* (%)	29	10 (34)					
Psychiatric medication, *n* (%)	29						
Mood stabilizers		29 (100)					
Antipsychotics		3 (10)					
Antidepressants		13 (45)					
Anxiolytics		3 (10)					
Hypnotics		1 (3)					
Illness duration^a^ in years, *M* (SD)	29	22.6 (11.2)					
Number of depressive episodes, *M* (SD)	29	12.8 (7.5)					
Number of (hypo)manic episodes, *M* (SD)	29	8.8 (6.4)					

*t*_y_ = *t* value based on Yuen–Welch test

^a^Variable is normally distributed

### Theory of mind performance

It was hypothesized that euthymic bipolar patients would perform worse than healthy controls on the MSAT and the RMET. One-tailed *t* tests were performed to test these predictions. Furthermore, it was hypothesized that eBPs and HCs would differ in their time taken to complete the sequencing part of the MSAT and to complete the RMET. These hypotheses were tested with two-tailed *t* tests. In this group of variables, four comparisons per variable were computed and the level of significance was Bonferroni-adjusted and set to *α* = .013. Contrary to our hypotheses, all group differences were statistically nonsignificant. On average, HCs performed better than eBPs on the MSAT, with the group difference representing a small effect size. A small effect size favoring patients was found for RMET performance. An effect size in the small range was also found for visual cognitive ToM sequencing time, where patients were slower than HCs. Patients were also slower to complete the Eyes Test, with group differences indicating a large effect. On the MSAT, 31 % of patients and 34 % of controls achieved a score of 59 (maximum) or 58 points, indicating a ceiling effect for that measure. Figures are presented in Table 2.

**Table 2:** Group comparisons for ToM and neurocognition

	Bipolar patients		Healthy controls		Statistics					
	*n*	*M* (SD)	*n*	*M* (SD)	*t*	*t*_y_	*df*	*p*	*d*	*ξ*
TAP: tonic alertness (RT)	29	270.9 (71.4)	29	257.2 (47.9)	0.86	0.85	48.97 28.82	.198 .201	0.22	0.16
TAP: phasic alertness (RT)	29	270.5 (72.7)	29	268.4 (60.5)	0.12	0.13	54.21 30.25	.453 .449	0.03	0.02
TAP: visual divided attention (RT)	29	792.4 (125.5)	29	820.1 (132.8)	0.82	0.06	55.82 33.50	.791 .526	0.21	0.01
TAP: auditory divided attention (RT)	29	620.0 (111.6)	29	575.3 (90.7)	1.67	1.32	55.75 29.04	.050 .100	0.44	0.25
TAP: divided attention (number of lapses)	29	1.4 (1.6)	29	2.0 (2.2)	1.15	1.12	50.53 34.37	.872 .865	0.30	0.19
TAP: go/no-go (RT)	33	435.1 (86.6)	29	398.6 (69.4)	1.77	1.96	53.45 31.07	.041 .030	0.46	0.41
WMS-R: digit span forward (total of correct trials)^a,b^	29	8.48 (2.0)	29	8.1 (2.2)	0.75	0.60	55.53 36.00	.773 .724	0.20	0.11
WMS-R: digit span backward (total correct trials)	29	6.9 (2.3)	29	6.9 (2.1)	0.06	0.49	55.30 35.89	.524 .315	0.02	0.09
VLMT: total score trials 1–5^a,b^	29	51.7 (9.7)	29	54.0 (9.3)	0.92	0.43	55.89 35.49	.180 .334	0.24	0.08
VLMT: trial 5 minus trial 7 (difference of words remembered)	29	2.0 (1.9)	29	1.3 (1.4)	1.50	1.45	52.24 29.25	.070 .079	0.39	0.28
VLMT: trial W minus F (correct minus errors)	29	12.8 (2.3)	29	13.4 (2.2)	1.00	1.14	55.90 35.99	.160 .262	0.26	0.21
TAP: go/no-go (number of errors)	29	1.0 (1.6)	29	0.6 (0.8)	1.24	0.78	42.10 35.97	.111 .221	0.33	0.14
Stroop (completion time)	29	84.0 (18.6)	29	75.0 (18.2)	1.86	2.50	55.98 35.33	.034 .009	0.49	0.41
RWT: subtest S-words (total correct responses)^a,b^	29	14.1 (4.6)	29	16.4 (4.3)	1.97	1.97	55.69 36.00	.027 .030	0.52	0.34
RWT: subtest animals (total correct responses)^a^	29	23.4 (5.7)	29	26.3 (5.1)	2.10	1.47	55.15 35.96	.021 .070	0.55	0.28
LPS3 (total correct items)	29	27.3 (6.2)	29	27.3 (6.6)	0.02	0.03	55.82 30.39	.492 .977	0.01	0.01
MWBT (total correct items)^a,b^	29	30.4 (305)	29	29.8 (3.9)	0.64	0.59	55.18 35.75	.736 .721	0.17	0.12
MSAT (total score)	29	53.3 (5.0)	29	54.3 (5.2)	0.72	1.06	55.94 34.55	.240 .851	0.19	0.19
MSAT sequencing time (sum completion time trials 1–6, seconds)	29	166.5 (75.3)	29	129.1 (46.1)	2.28	1.40	46.40 35.75	.027 .169	0.60	0.28
Reading the Mind in the Eyes Test (total correct items)^a^	29	25.1 (4.0)	28	23.6 (4.0)	1.42	1.35	54.93 34.87	.919 .910	0.38	0.27
Eyes time (completion time, minutes)	26	10.3 (3.7)	20	8.2 (1.7)	2.52	2.20	31.10 25.74	.016 .037	0.52	0.51

*RT* reaction time (mean median reaction times)

^a^Variable is normally distributed

^b^No outliers in either group

### Neurocognitive functioning

As a total of 17 correlations were calculated, the alpha-level for statistical significance was Bonferroni-adjusted and set to *α* = .003. As evidence points to stable neurocognitive deficits in bipolar patients, we hypothesized that eBPs would perform worse than HCs on our measures of neurocognition. One-tailed *t* tests were performed to test these hypotheses.

All group differences were statistically nonsignificant. In terms of the effect sizes, no effects were observed for differences between eBPs and HCs in phasic alertness, visual divided attention, working memory, reasoning abilities and verbal intelligence. Small effect-sizes favoring controls were observed for tonic attention, auditory divided attention, selective attention, verbal learning, verbal consolidation, verbal recognition, cognitive inhibition as measured by the Go/No-go error rate, and cognitive inhibition as measured by the Stroop Task. Effect sizes in the small range favoring patients were found for general divided attention, and short-term memory. Effect sizes in the medium range favoring controls were found for lexical and semantic verbal fluency. Figures are presented in Table 2.

### Correlations

In the bipolar group, Kendall’s tau correlation coefficients were calculated as robust statistics to determine the associations between ToM performance variables (MSAT and RMET sum scores) and neurocognitive measures (all measures displayed in Table 2), and between ToM performance variables and clinical measures (BDI total score, ASRM total score, HAMD total score, YMRS total score, years of illness duration, number of depressive episodes, and number of (hypo)manic episodes). For each ToM variable, 24 correlation coefficients were computed, and, the family-wise alpha-level was set to *p* = .002. In terms of effect sizes, weak negative correlations were observed between visual cognitive ToM performance and tonic alertness (*r*_*τ*_ = −.20, *p* = .135), visual divided attention (*r*_*τ*_ = −.22, *p* = .10), general divided attention (*r*_*τ*_ = −.23, *p* = .113), and verbal consolidation (*r*_*τ*_ = −.21, *p* = .141). Moderate positive correlations were found between visual cognitive ToM performance and verbal recognition (*r*_*τ*_ = .45, *p* = .002), reasoning abilities (*r*_*τ*_ = .42, *p* = .003), and verbal learning (*r*_*τ*_ = .45, *p* = .001), the latter reaching statistical significance. All reported positive and negative correlations indicated that a higher performance in one test was associated with higher performance on the other tests.

Visual affective ToM performance showed a weak negative correlation with selective attention (*r*_*τ*_ = −.23, *p* = .096), and a weak positive correlation with verbal learning (*r*_*τ*_ = .25, *p* = .075). A moderate positive correlation was found between RMET performance and verbal recognition (*r*_*τ*_ = .35, *p* = .016) and with reasoning abilities (*r*_*τ*_ = .30, *p* = .032). A moderate negative correlation was found between RMET performance and tonic alertness (*r*_*τ*_ = −.38, *p* = .006), and with phasic alertness (*r*_*τ*_ = −.34, *p* = .013). Again, all reported positive and negative correlations indicated that a higher performance in one test was correlated with a higher performance on others.

Concerning the clinical variables, a weak positive correlation was observed between clinician-rated depressive symptoms (HAMD) and RMET performance (*r*_*τ*_ = .21, *p* = .136), where having more symptoms was correlated with a higher number of correctly answered items. Number of illness years was weakly and negatively correlated with the MSAT sum score (*r*_*τ*_ = −.25, *p* = .063), indicating a lower performance over the course of years living with the illness. No other correlations above 0.20 were observed between clinical variables and cognitive or affective ToM performance.

As the groups were not matched for age, Kendall’s tau correlation coefficients were calculated between age and the primary outcome measures MSAT sum score and RMET sum score within each group separately. A total of two correlations were calculated per group; so, the alpha-level for statistical significance was Bonferroni-adjusted and set to *α* = .025. In the patient group, age was negatively correlated in a statistically significant way with visual cognitive ToM performance (*r*_*τ*_ = −.31, *p* = .023). The negative correlation found between age and visual affective ToM was also in the moderate range but did not reach statistical significance (*r*_*τ*_ = −.30, *p* = .031). Both correlations indicated a lower performance with increasing age. In the group of HCs, age was not correlated with either variable.

## Discussion

A recent meta-analyses indicates the occurrence of a ToM deficit in bipolar patients across all mood states, with remitted or subsyndromal patients showing stable modest deficits, albeit less severe than during acute episodes (Bora et al. 2016). Nevertheless, the variability in effect sizes between studies is large and the role of possible confounding factors such as neurocognitive functions and clinical variables has not been established conclusively. Also, differential patterns of impairment might exist depending on task area (cognitive or affective) and task modality (verbal or visual). In the present study, we aimed to exclusively examine the performance of euthymic bipolar patients in visual cognitive and affective theory of mind in comparison to healthy subjects, as deficits in those domains have been less consistently shown for remitted than for acute patients (Kerr et al. 2003; Ioannidi et al. 2015; Olley et al. 2005). Contrary to our hypotheses, patients did not differ in a statistically significant way from controls on our measures of visual cognitive and visual affective ToM. Also in disagreement with our hypotheses, we did not find statistically significant differences between patients and controls regarding their time taken to complete either of the ToM tasks. In terms of effect sizes, our findings were also below the effect sizes reported in recent meta-analyses. While Samamé et al. (2015) have reported effect sizes in the small-to-medium range for cognitive ToM, and Bora et al. (2016) have reported effect sizes in the moderate range pooling across ToM tasks, we observed only a small effect in favor of controls on the performance of our cognitive ToM task. On our visual affective ToM task, there was even a small effect favoring the performance of patients. With respect to ToM task completion time, we found controls to be faster than patients with a small effect size on the cognitive ToM task. A large effect in favor of controls was found for the time taken to complete the affective ToM task.

In direct comparison to studies that also used a cartoon task to measure visual cognitive ToM, our finding is in contrast with the results of the study by Wolf et al. (2010) that found a statistically significant difference between patients and controls, with a large effect in favor of healthy participants. On the other hand, our results are in line with findings by Kerr et al. (2003) and Olley et al. (2005) that reported similar performance for patients and controls. Importantly, patients in our study and in the study by Olley et al. presented with a high level of neurocognitive functioning, whereas they showed pronounced deficits in executive functioning relative to controls in the study by Wolf and colleagues. In our study, none of the group differences on neurocognitive measures were statistically significant. Further, our effect sizes were on the lower edge of or below the effect sizes reported in the previous literature. Notably, we did not find the typically reported medium-to-large or large effect sizes in the domains of verbal memory and cognitive inhibition, which are currently discussed as possible cognitive endophenotypes of BD (Fountoulakis 2015). Furthermore, the remitted group in the study by Wolf et al. showed slightly elevated levels of subsyndromal symptoms; in particular a higher level of manic symptoms. In the meta-regression analysis performed by Bora et al. (2016), clinician-rated manic symptoms were significantly related to the severity of ToM deficits. Moreover, the patients in the study by Wolf et al. were remitted in the course of the last 4 weeks prior to testing, while they had to be remitted for at least 4 weeks in the study by Olley et al., and for at least 6 weeks in our study. It is possible that not only the decrease of symptoms, but also the time passed since remission is important for the recovery of neurocognitive and social-cognitive abilities. In a longitudinal study by Volkert et al. (2016) that employed a within-subjects pre-post design, cognitive performance in bipolar patients had considerably improved after 3 months of euthymia.

Our finding of patients performing in line with controls on the RMET is in contrast with effect sizes in the small-to-medium range favoring controls reported for this visual affective ToM task in the abovementioned meta-analyses. Nonetheless, some studies that directly compared affective ToM and cognitive ToM in eBPs have found differential patterns of impairment, with affective ToM abilities being preserved or less affected than cognitive ToM abilities (Samamé et al. 2015; Montag et al. 2010; Barrera et al. 2013; Shamay-Tsoory et al. 2009). Furthermore, as with cognitive ToM, neurocognitive functioning seems to be related to affective ToM performance, and this may, therefore, partly explain the diversity of findings. This hypothesis is supported by evidence from a recent study that has suggested the existence of several neurocognitive subgroups within the population of bipolar patients (Burdick et al. 2014). Importantly, the group of patients, which was on the same level of neurocognitive functioning as the HCs, outperformed the HCs on a measure of affective social cognition, while the other two patient subgroups performed worse than the controls on that measure. This finding suggests that in a subgroup of bipolar patients with preserved neurocognitive functioning, affective ToM abilities might even be elevated compared to the general population.

Considering that we chose to measure cognitive and affective ToM abilities based on visual material, our findings do correspond with results of the study by Olley et al. (2005), where a ToM deficit was observed in the verbal cognitive ToM task, but not in the visual cognitive ToM task. This pattern has also been found in unaffected first-degree adult relatives (FDRs) of individuals with BD (Reynolds et al. 2014), where FDRs performed worse than HCs on a cognitively demanding verbal ToM task, but showed no deficits on either the cognitive or affective visual ToM tasks. Thus, our results also support the hypothesis of these authors that ToM deficits in BD might represent a modality-specific disturbance, with visual social perceptual skills being preserved or less affected than verbal social cognitive skills.

Through the comparison of studies with diverging results on ToM in BD, it has become apparent that basic neurocognitive functioning might be decisive for ToM performance. In our study, only the correlation between performance on the MSAT and verbal learning reached statistical significance. Nonetheless, correlations between both ToM tasks and several measures of verbal memory, attention and reasoning were in the small-to-moderate range and could indicate existing relationships and practical significance.

Results concerning the effect of illness burden and subsyndromal symptoms on ToM abilities are more ambiguous. In our sample, neither variables of illness burden nor measures of subsyndromal symptoms were significantly related to ToM performance. However, this finding might be due to the low level of current symptoms and the high level of neurocognitive functioning and ToM performance in our sample. Interestingly, ToM performance was negatively correlated with age in the bipolar group but not in the control group. In the study by Wolf et al. (2010), age was also found to correlate negatively with performance on ToM tasks. An explanation for this finding might be that age by itself represents an indicator of illness severity that bundles factors such as illness duration and number of episodes which might be related to a decline in cognitive abilities. Yet, there is also evidence pointing to a null relationship between age and ToM abilities and BPs (Donohoe et al. 2012; Inoue et al. 2004), and the effect we found could be spurious. In sum, the correlations reported in the current study are in line with the literature where the link between ToM performance and basic neurocognitive functions has more consistently been emphasized over and above the relationship between ToM performance and clinical variables (Mitchell and Young 2016; Bora et al. 2016).

Yet, neurocognitive functions themselves seem to be vulnerable to clinical variables which might, therefore, also be relevant for social cognition, albeit indirectly. Preliminary findings indicate that illness severity factors such as age at illness onset, number of episodes (especially manic episodes) and absence of remission could be related to a progressive neurocognitive decline (Fountoulakis 2015). Similar findings have emerged from recent studies that have suggested the existence of several neurocognitive subgroups within the population of BPs (Burdick et al. 2014; Martino et al. 2014). In one of these studies, neurocognitive impairment was related to the total number of affective episodes (Burdick et al. 2014). Another study showed a higher number of hospitalizations in the subgroup of cognitively impaired BPs compared to the subgroup of cognitively preserved patients (Martino et al. 2014). In a study by Volkert et al. (2014), where BPs were divided into two subgroups according to their neurocognitive performance, the group that showed deficits in at least one neurocognitive domain reported more sub-threshold depressive symptoms, more sleep disturbances, and, more often, a comorbid anxiety disorder compared to the group that was on the same level of neurocognitive functioning as HCs. Our sample of patients that did not show deficits in neurocognitive and social-cognitive functioning might, thus, be interpreted as representing the subgroup of BPs with preserved neurocognitive functions. This sample composition might be due to our strict criteria of euthymia and time elapsed since remission, or due to effective prophylactic treatment (Pfennig et al. 2014). What may be even more important than the clinical stability of our patients is their relatively high level of education: While there is no evidence to support this hypothesis to date, it is plausible that education levels may be related to neurocognitive performance.

In summary, our study supports the notion that visual cognitive and visual affective ToM abilities are not impaired in bipolar patients with a high level of neurocognitive functioning. However, our results indicate that patients might take more time to complete ToM tasks which by itself could be relevant for psychosocial functioning. It might be that not only the level of performance is relevant to social communication, but also the speed at which inferences about mental states can be drawn. Future studies are warranted to investigate the different facets of ToM abilities in neurocognitive subgroups of BD, and to identify factors associated with neurocognitive and social-cognitive impairments in BD, e.g., clinical variables like single symptoms and recovery time. In view of the variability of findings reported across studies, it seems too early to draw conclusions about the stability of ToM deficits in BD.

Several limitations of our study have to be considered. First, as we only assessed measures of visual cognitive and affective ToM, no comparisons could be made with performance on measures of verbal cognitive and affective ToM. Furthermore, our measure of visual cognitive ToM showed a ceiling effect which might mean that the task was too easy to detect an effect. Further limitations to our study are the small sample size which is associated with low statistical power, and the possible influences of psychotropic medication on the performance of patients. Also, our patient sample was characterized by a higher level of neurocognitive functioning than is often reported in the literature. Moreover, our samples were not matched for age. Yet, as age was negatively correlated with ToM performance in the bipolar sample, we considered it unlikely that the result of patients performing similarly to controls on neurocognitive and ToM tasks should be due to their higher age. Taken together, the current study controlled for a range of important variables and showed the importance of considering basic neurocognitive and clinical variables when examining ToM abilities in BD. Future research should address the problems outlined above by developing ToM tasks that are difficult enough to produce sufficient variance and by examining larger samples.

## Abbreviations

ToM: theory of mind
BD: bipolar disorder(s)
BPs: bipolar patients
eBPs: euthymic bipolar patients
HC: healthy controls
FDRs: unaffected first-degree adult relatives
MSAT: Mental State Attribution Task
RMET: Reading the Mind in the Eyes Test
TAP: Test of Attentional Performance
HAMD: Hamilton Depression Scale
YMRS: Young Mania Rating Scale
BDI: Beck Depression Inventory
ASRM: Altman Self-Rating Mania Scale
MINI: Mini International Neuropsychiatric Interview
ICD-10: International Statistical Classification of Diseases and Related Health Problems
